# Complete genome sequence for the thermoacidophilic archaeon *Metallosphaera sedula* (DSM:5348)

**DOI:** 10.1128/mra.01228-23

**Published:** 2024-02-08

**Authors:** Mohamad J. H. Manesh, Ryan G. Bing, Daniel J. Willard, Robert M. Kelly

**Affiliations:** 1 Department of Chemical and Biomolecular Engineering, North Carolina State University, Raleigh, North Carolina, USA; Portland State University, Portland, Oregon, United States

**Keywords:** *Metallosphaera sedula*, thermoacidophile, archeaon

## Abstract

The complete genome sequence of the thermoacidophilic archaeon *Metallosphaera sedula* (DSM 5348) is reported here. *M. sedula*, originally isolated from a volcanic field in Italy, is a prolific iron-oxidizing archaeon with applications in bioleaching of sulfide minerals.

## ANNOUNCEMENT

*Metallosphaera sedula* is an obligate aerobe, Chemolithoautotroph/heterotroph, isolated from a solfataric field in Italy, with optimal growth at pH of 2.0 and 73°C (1, 2). It was assigned to the new genus *Metallosphaera* in 1989 and is described as an iron-oxidizing archaea with applications in biomining of sulfide minerals (2, 3). It can also be grown on meteorites (4). The previous *M*. sedula reference genome was removed from NCBI due to inability to verify source material. The closed genome sequence of *M. sedula* DSM 5348 is reported here, consisting of 2,190,631 bp.

*M. sedula* was obtained from DSMZ and grown from −80°C freezer stocks at 70°C in modified DSM88 medium. NEB Monarch Genomic DNA Purification Kit (New England Biolabs, USA) was used to extract and purify genomic DNA from liquid cultures. The 1× dsDNA HS Assay Kit (Invitrogen, Q33231) was used to quantify DNA on a Qubit 4.0 fluorimeter. Oxford Nanopore Technologies (UK) Native Barcoding Kit (SQK-NBD112-24) was used for DNA barcoding, and Oxford Nanopore Technologies (UK) R9.4.1 flow cell (FLO-MIN106D) on a MinION Mk1B was used for DNA sequencing without size selection Sequencing was carried out for 72 h for the library using MinKNOW v22.12.7, while Guppy v6.4.6 was used for live high-accuracy GPU base calling and quality verification of the run, respectively.

Guppy base-calling workflow was used to trim the long reads initially, and NanoFilt v2.8.0 (5) was used to filter the reads, with read quality and length cutoffs set at 10 and 1,000, respectively. The ONT long reads were then used to generate a circularized contig for each strain using Flye assembler (v2.9.1-b1780) (6). Rotation of the assembled genome to start at the *dnaA* gene was carried out using the fixstart command in Circlator v1.5.5 (7). GraphMap v0.5.2 (8) was used for mapping the reads, SAMtools v1.16.1 (9) was used for indexing and sorting the mapped reads, and Pilon v1.24 (10) and Medaka v1.11.1 (Oxford Nanopore Technologies, UK) were used for error correction and polishing of the assemblies. Error correction and polishing were done using the trimmed and filtered reads. QUAST v5.2.0 (11), Pilon (10), and CheckM v1.2.2 (12) were used to generate quality and statistics reports for the finalized assemblies. Annotation of final assemblies was carried out using PGAP2023-10-03.build7061 . All software was used with default parameters unless specified otherwise. A summary of assembly statistics is shown in Table 1.

**TABLE 1:** Statistics for *M. sedula* genome assembly

	Metallosphaera sedula
Genome Accession ID	CP139956
BioProject	PRJNA1046088
BioSample	SAMN38472205
Sequence Read Archive	SRR27048297
# of Reads	126,414
Total Reads	571,623,454
Read n50	7,468
Genome Size	2,190,631
GC Content	46.22%
Read Coverage	240
Completeness	100.00
Contamination	0.00
# of Predicted Genes	2,367

## Data Availability

To access the data, the accession numbers, including raw reads SRA, BioSample, BioProject, and Genome, used in this project, previously deposited at NCBI, are provided in Table 1.
